# Improvement of oral health knowledge and behavior of diabetic patients: an interventional study using the social media

**DOI:** 10.1186/s12903-023-03007-w

**Published:** 2023-06-03

**Authors:** Atousa Haghdoost, Soheila Bakhshandeh, Sajjad Tohidi, Zahra Ghorbani, Mahshid Namdari

**Affiliations:** 1 Dental Research Center, Research Institute of Dental Sciences, School of Dentistry, Shahid Beheshti University of Medical Sciences, Tehran, Iran; 2 Department of Community Oral Health, School of Dentistry, Shahid Beheshti University of Medical Sciences, Tehran, Iran; 3 Department of Endodontics, School of Dentistry, Qazvin University of Medical Science, Qazvin, Iran; 4 Department of Biostatistics, School of Allied Medical Sciences, Shahid Beheshti University of Medical Sciences, Tehran, Iran

**Keywords:** Oral health, Diabetes Mellitus, Knowledge, Educational intervention, Social media

## Abstract

**Background:**

Diabetic patients are not often aware of relationship between diabetes mellitus (DM) and periodontal diseases, and the researchers recommend further knowledge enhancement of diabetic patients in this regard. This study aimed to enhance oral health knowledge of diabetic adults via an educational intervention.

**Methods:**

In this interventional study, three private offices of endocrinologists specialized in treatment of DM were selected for the recruitment of participants. In total, 120 diabetic adults (40 from each office) took part in an educational intervention in three groups (patients from each office made up one group): (I) physician-aid, (II) researcher-aid, and (III) social media. In group (I), participants received educational materials (brochure and CD) from their endocrinologist, in group (II) participants received educational materials from researcher. Group (III) joining an educational group in WhatsApp for 3 months. A self-reported standard questionnaire was filled out by the patients before, and after the intervention to assess oral health knowledge. Data were analyzed by SPSS version 21 using independent t-test, Mann-Whitney test, Chi-square test, and ANCOVA.

**Results:**

The mean oral health knowledge score increased in all three groups after the educational interventions (P < 0.001); the highest increase occurred in the social media group. Toothbrushing twice daily or more had the greatest improvement in the physician-aid group compared with the other two groups (P < 0.001). The greatest improvement in dental flossing once daily or more occurred in the social media group (P = 0.01). The mean level of the hemoglobin A1c (HbA1c) decreased in all three groups, but not significantly (P = 0.83).

**Conclusion:**

The results showed that educational interventions enhance oral health knowledge, and improve the behavior of diabetic adults. The education via the social media can be an efficient method for knowledge enhancement of diabetic patients.

## Introduction

Diabetes mellitus (DM) which is a metabolic disorder is a global public health dilemma characterized by hyperglycemia [1]. In accordance with American Diabetes Association guidelines from 2016, DM is diagnosed when hemoglobin A1c (HbA1c) levels are more than 6.5% and HbA1c values below 7% are regarded as treatment objectives for adults [2, 3]. The World Diabetes Federation projects that there will be 693 million diabetes patients between the ages of 18 and 99 in 2045. [4]. It was estimated that there will be approximately 9.2 million diabetic patients in Iran by 2030 [5].

Although there is no specific oral lesion related to DM, long-term hyperglycemia can have oral manifestations, such as oral mucosal burning sensation, xerostomia, dental caries, and periodontal disease (gingivitis, and periodontitis), and may even lead to premature tooth loss [6, 7]. Periodontal disease is among the oral complications of DM, which is aggravated by hyperglycemia. Moreover, the systemic inflammation in terms of periodontitis decreases the blood glucose level in diabetic patients. Thus, there appears to be a mutual relationship between DM, and periodontal disease [8–10].

Precise oral hygiene can decrease oral inflammation, and decelerate periodontal degradation in diabetic patients. Regular toothbrushing, and dental visits can decrease the prevalence of periodontitis by 34% and 32%, respectively. In contrast, poor oral hygiene can significantly increase the risk of periodontitis by 2 to 5 times [11, 12].

Oral hygiene instruction to the public and high-risk groups and enhancement of their health literacy are among the cornerstones of disease control [13]. Data suggests that people with diabetes are not adequately informed about their increased risk of oral disorders and the link between DM and periodontal disease. Thus, researchers advise diabetes patients to increase their knowledge [14]. It was documented that oral hygiene instruction in the combination with conventional dental treatments can have beneficial effects on general health [15]. Health promotion refers to the empowerment of individuals and the society to better control factors that affect health, and promote the health status as such. One of the principles of Ottawa Charter is to promote the health and individual skills. Developing skills, such as tooth cleaning and oral hygiene can enhance knowledge, reinforce hygienic behaviors, and develop a sense of participation, cooperation, and support [16, 17].

Internet and advances in digital technology have revolutionized our communication paths. Social media are among such technologies that have gained increasing popularity in the recent years, and include a series of Internet-based applications that enable generation and sharing of contents with users [18]. Social media are good instruments for promoting public health because they are precise, affordable, and help people retain information [19]. Patients utilize social media as a resource to learn about a particular ailment, engage and communicate with others quickly and effectively, and share medical information with other patients experiencing the same condition [20–22].

Social media are a valuable source for diabetic patients to improve their self-management skills. The patients with the same experiences and conditions can easily connect with each other and communicate via social media, and share their knowledge with peers and support each other [23]. Nonetheless, insufficient evidence exists to support the role of social media in improvement of self-management behaviors, and health outcomes in diabetic patients [24]. Lifestyle changes, such as dietary changes, exercise, blood glucose monitoring, online education, support by peers, and communication with patients and healthcare specialists can all be enhanced through the social networks [21, 23, 25].

Considering the extensive use of smartphones, such novel technologies can provide new opportunities for users, which can be used as affordable, fast, and powerful tools of communication and data sharing [26]. Considering high number of users, social networks were proposed as effective educational tools, which can be used to promote preventive measures and cause behavior change [20, 27–29].

We decided it was vital to undertake a research to evaluate the impact of educational interventions using social media on the oral health awareness of diabetic adults in Tehran given the significance that social media plays in modern life and the daily access that everyone has to them. We hypothesized that educational intervention via the social media would be effective to improve oral health knowledge, behaviors and HbA1c level of patients with diabetes.

## Materials and methods

### Study design and sample

In this interventional pre-post study, three private offices of endocrinologists specialized in treating DM patients were selected from different districts of Tehran in August 2019. The participants were divided into three groups: physician assistance, researcher assistance, and social media. Individual randomization was not feasible due to the risk of data tampering (it was possible that patients in the same office share their brochures or information with each other). As a result, participants chosen from each office were put into a single group. Based on a previous study [30], the mean knowledge score of diabetic patients was considered to be 5.26 with a standard deviation of 1.9. Considering 35% improvement in the mean knowledge score, alpha = 0.05, and beta = 0.15 in two groups, the sample size was calculated to be 21 in each group. Considering the presence of 3 groups, and the possibility of loss to follow-up to be 25%, 40 patients were recruited for each group using following formulas:

$$n=n\times \sqrt{K-1}$$

$$n=21\times \sqrt{3-1}=21\times 1/4\cong 30$$

A total of 120 patients (40 from each office) were enrolled by convenience sampling. Adults between 18 and 50 years old, who had at least two healthy functional teeth, were included. The patients were briefed about study objectives, and were ensured about the confidentiality of their information. Illiterate patients and pregnant women were excluded from the study. The subjects provided written informed consent. The permission form includes information about the study’s objectives, investigators, privacy concerns, and a statement about participants’ voluntary involvement. Eligible participants were enrolled such that the three groups were similar due to age and gender. For instance, in each group, 5–7 females between 18 and 30 years were required. Sampling was continued until the sample size was reached.

### Questionnaire face validity

A self-reported structured standard questionnaire [30] was used for data collection. The process of translation, and back translation was conducted for it. For face validity, evaluation was made from the consensus of eight experts (dental public health specialists and periodontists). The questionnaire was subjected to expert’s face validity analysis, and Content Validity Indices were calculated higher than 0.79. In a pilot research involving 40 patients visiting a private clinic in Tehran, the questionnaire’s face validity was evaluated by the users based on the questions’ organization, syntax, clarity, and relevancy. Based on observations collected from the participants, linguistic-cultural adjustments were made to any items considered hard to understand.

The first part of the questionnaire included demographic information, the second part included information regarding DM, the third part was about self-care behaviors, and the final part included 10 oral health knowledge questions with answer choices of correct (1 score), wrong (0 score), and I do not know (0 score). The sum of scores was calculated for each patient (Table 1).

**Table 1:** Questions about oral self-care, oral health practice, and oral health knowledge of diabetic adults

**Oral self-care and oral health practice:** - Are you aware of oral self-care and oral diseases? Yes (I have some information, but it is not sufficient) /No (I have not received such information/I do not know) • Where did you acquire your information? physician/dentist or dental assistant/publications on DM*/Internet/the media/social media - Are you aware of the effect of periodontal disease on DM? Yes/No • Where did you acquire your information? physician/dentist or dental assistant/publications on DM*/Internet/the media/social media - How often do you brush your teeth? less than twice a day (rarely once a week/several times a week/once daily) /twice daily or more (more than once a day) - How often do you floss? less than once a day (rarely or never/several times a week) /once a day or more
**Knowledge about oral health and major effects of DM on it**: 1. Gingival bleeding during toothbrushing is normal. 2. Tobacco use increases mouth dryness. 3. Redness and swelling are the symptoms of gum disease. 4. Oral health can affect general health. 5. Dental calculus can form subgingivally as well. 6. Food residues and bacteria are main causes of formation of dental calculus. 7. Microbial plaque is the main cause of gum disease. 8. Even in case of gingival health, regular tooth cleaning is imperative. 9. Impairment of sense of taste is the most important complication of DM. 10. Diabetic patients should visit a dentist at least every 6 months.

* Diabetes mellitus

### Data collection and intervention

After the completion of questionnaire, the patients were visited by an endocrinologist. In the physician-aid group, after the completion of questionnaire, the endocrinologist first emphasized on significance of oral health, and oral hygiene, and its pivotal role in general health and control of DM. Then, under the guidance of experts including psychologists, oral medicine and community dentistry specialists, and using the most recent scientific resources, the researcher gave the patients an educational brochure and an educational film on a CD that had been created in accordance with international standards and under the supervision of experts. The endocrinologist strictly emphasized on careful reading of brochure and watching the film. The educational content included oral diseases in DM patients, oral complications of DM, description of periodontal diseases, correlation of oral diseases, and DM, prevention of oral complications of DM, advantages and reasons of regular dental visits, instruction of oral self-care behaviors (such as toothbrushing, dental flossing, and using mouthwash), and medical considerations in dental office. In researcher-aid group, after completion of the questionnaire, the researcher first emphasized on significance of oral health, and oral hygiene and its pivotal role in general health and control of DM using the same sentences as those used for physician-aid group. Then, he/she provided the patients with an educational brochure, and an educational film on a CD and strictly emphasized on careful reading of the brochure and watching the film. In the social media group, after the completion of the questionnaire, the researcher invited the patients to join an educational group in WhatsApp for 3 months, and asked them to pay attention to oral heath educational messages, and recommended oral health self-care behaviors that are uploaded in the group. The patients joined the group by the researcher, and educational topics of the brochure and film were gradually uploaded in the group every 3 days for a total duration of 3 months.

After 3 months, the researcher contacted the patients, and asked questions of the questionnaire from them over the phone. The first part asked for the most recent HbA1c level of patients. The second section asked for self-care practices, and the last section included 10 oral health-related knowledge questions. In the event of a non-response, each patient was only approached a maximum of three times. First, we questioned patients in the physician-aid and researcher-aid groups whether they had read the pamphlet and listened to the CD. Moreover, all patients in the social media group were checked by the researcher to make sure that they read the contents since WhatsApp has this feature to ensure that each individual message was seen. In case of not seeing the messages, the patient would be excluded from the study. The patients had regular visits to the office every 3 months to consult their HbA1c test results with their endocrinologist.

**Fig. 1:**
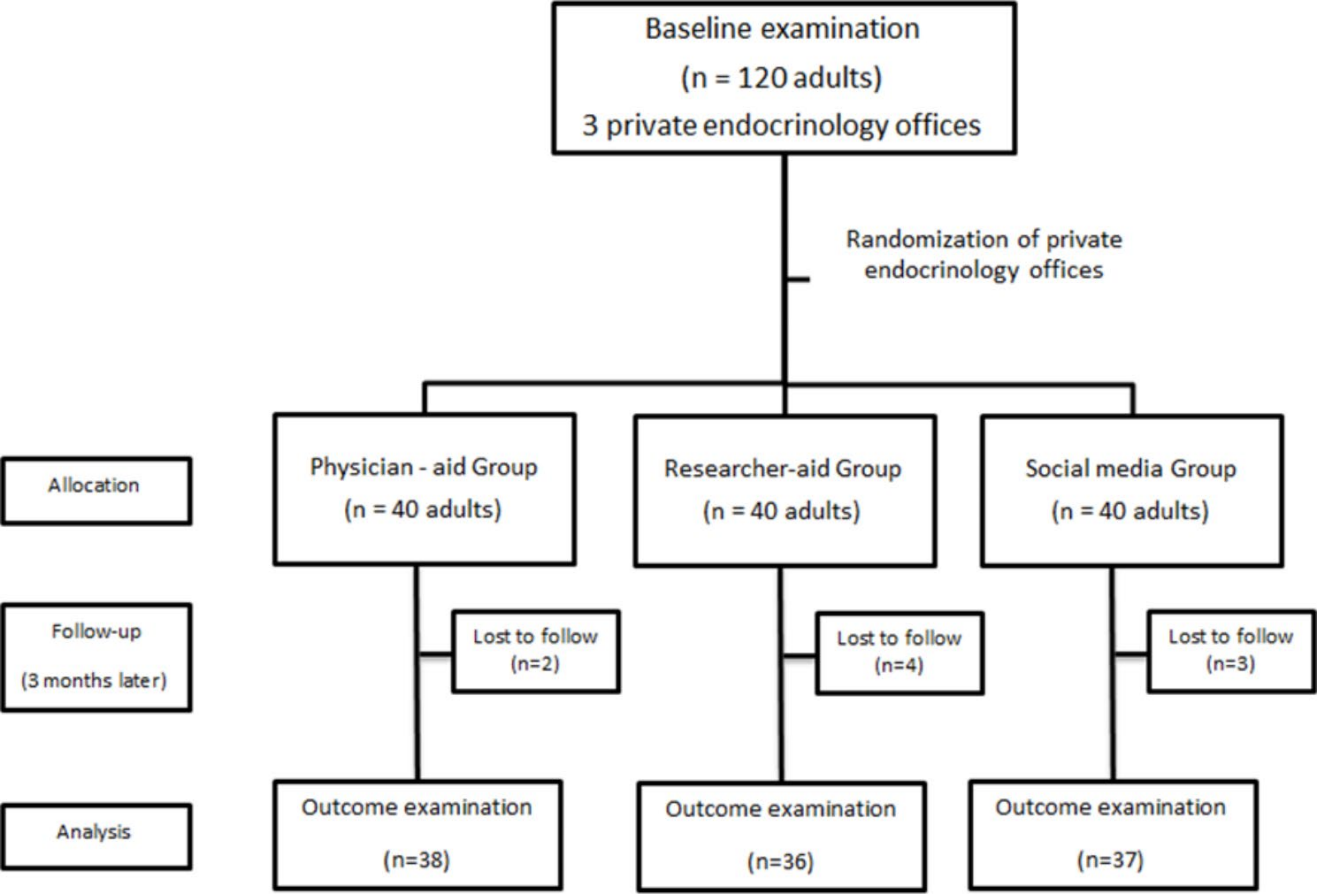
Flowchart of patient selection and allocation

Two patients in the physician-aid group and 3 patients in the researcher-aid group were excluded since they could not be contacted after 3 attempts. Furthermore, another patient was excluded from the researcher-aid group since he did not read the brochure and did not watch the clip. In the social media group, 1 patient was excluded in terms of complete recovery and another one due to unwillingness to remain in the study. Moreover, another patient was excluded since he could not be reached after 3 attempts (Fig. 1).

### Statistical analyses

Data were analyzed by SPSS version 21. Descriptive measures, such as mean and standard deviation were reported. Chi-square, Mann-Whitney, and independent t-tests were used for bivariate statistical analysis. Using ANCOVA to control the impact of mean baseline knowledge score and mean level of most recent HbA1c, the mean knowledge score and mean level of most recent HbA1c were compared across the three groups.

### Ethical issues

This intervention was approved by the ethics committee of Shahid Beheshti University of Medical Sciences (IR.SBMU.DRC.REC.1398.014).

## Results

Among 120 participants with a mean age of 35.8 ± 10.5 years, 63 (52.5%) were females and 57 (47.5%) were males. Among entire study population, 55% had type 1 DM, and 45% had type 2 DM. The mean level of most recent HbA1c was 7.4% (n = 117, standard deviation of 1.56%, range 4.9 to 13.2%).

No significant difference was found at baseline among three groups regarding the type of DM, history of DM, mean level of most recent HbA1c, and oral self-care behaviors (Table 2). 81% of the population surveyed said they were adequately informed about dental care to prevent oral diseases; 30% said they got that information from the internet, 27% from their dentist or dental assistant, and 27% from other media, including radio and television. Among the study population, only 30% were aware of the effect of periodontal disease on DM; out of which, 16.7% acquired these information from publications regarding DM and 11.7% from their endocrinologist.

**Table 2:** Frequency percentage of self-care behaviors and DM* status of patients at baseline (n = 120)

Variables			Physician-aid (N = 40) N (%)	Researcher-aid (N = 40) N (%)	Social media (N = 40) N (%)	p-value**
Information about diabetes	Type of diabetes	Type one	26 (65.0)	21 (52.5)	19 (47.5)	0.269
		Type two	14 (35.0)	19 (47.5)	21 (52.5)	
	Duration of diabetes	Under 7 years	8 (20.0)	15 (37.5)	18 (45.0)	0.134
		7–12 years	10 (25.0)	11 (27.5)	9 (22.5)	
		Over 12 years	22 (55.0)	14 (35.0)	13 (32.5)	
	Latest HbA1c^***^ level	< 7%	15 (37.5)	18 (45.0)	18 (45.0)	0.600
		> 7%	25 (62.5)	19 (47.5)	22 (55.0)	
		Non-response	0 (0.0)	3 (7.5)	0 (0.0)	
Self-care behaviors	Regular dental visits	Yes	10 (25.0)	11 (28.2)	6 (15.0)	0.342
		No	30 (75.0)	28 (71.8)	34 (85.0)	
		Non-response	0 (0.0)	1 (2.5)	0 (0.0)	
	Information about the effect of periodontal disease on DM	Yes	17 (42.5)	11 (27.5)	8 (20.0)	0.082
		No	23 (57.5)	29 (72.5)	32 (80.0)	
	Tooth brushing	Less than twice a day	29 (72.5)	24 (60.0)	25 (62.5)	0.463
		Twice a day and more	11 (27.5)	16 (40.0)	15 (37.5)	
	Flossing	Rarely	30 (75.0)	22 (55.0)	21 (52.5)	0.078
		Once a day or more	10 (25.0)	18 (45.0)	19 (47.5)	

* Diabetes mellitus

** Independent sample t-test

*** Hemoglobin A1c

At baseline, 22.5% had regular dental visits. 65% brushed their teeth less frequently than twice daily while 35% reported brushing twice a day or more; 60.8% reported brushing less than once a day, and 39.2% reported dental flossing once daily or more. The mean oral health knowledge score was 5.74 (standard deviation = 1.85, range 1–10) at baseline.

Of 111 patients after the intervention, 27.9% reported visiting a dentist in the past 3 months. After the intervention, 49.5% reported tooth brushing less than twice a day, and 50.5% reported tooth brushing twice a day or more. Furthermore, 44.1% reported brushing less than once a day, and 55.9% reported dental flossing once a day or more. After the intervention, the mean knowledge score reached to 8.07 (standard deviation = 1.23, range 5–10) among 111 patients.

### Effect of the intervention on knowledge

The mean oral health knowledge score at baseline was not significantly different among three groups (P = 0.74). The mean oral health knowledge score significantly increased in all three groups after the intervention, compared with baseline (P < 0.001). Maximum increase occurred in the social media group. After controlling for the effect of mean baseline oral health knowledge score by ANCOVA, the mean knowledge score was found to be significantly different among three groups after the intervention (P < 0.001). The mean final knowledge score in the researcher-aid group was significantly lower than that in the physician-aid (P = 0.02) and social media (P < 0.001) groups. The difference between physician-aid, and social media groups was not significant (P = 0.15). The three groups’ mean changes in oral health knowledge scores are shown in Table 3.

**Table 3:** Comparing the mean score of oral health knowledge at baseline and after the intervention (n = 111)

Groups	Observed mean knowledge score		Adjusted mean of knowledge score after intervention*	After intervention _ Baseline	
	Baseline	After intervention			
	Mean (SD)	Mean (SD)	Mean (SE)	Mean (SD)	p-value
Physician-aid	5.92 (1.71)	8.18 (1.20)	8.13 (0.16)	2.26 (1.50)	< 0.001
Researcher-aid	5.61 (1.82)	7.44 (1.23)	7.48 (0.17)	1.83 (1.66)	< 0.001
Social media	5.68 (1.87)	8.57 (0.99)	8.59 (0.16)	2.89 (1.61)	< 0.001
p-value	0.74	< 0.001	< 0.001		

*Adjusted with baseline values by ANCOVA

### Effect of the intervention on behaviors

**Fig. 2:**
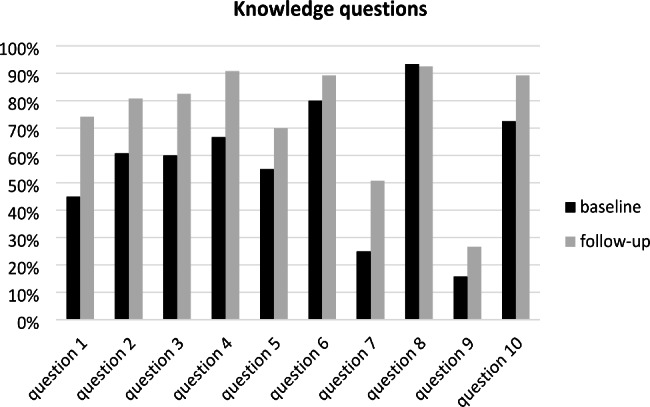
The percentage of correct responses to 10 knowledge questions before and after the intervention

Figure 2 displays the proportion of right answers to each of the 10 knowledge questions before and after the intervention. In total, the lowest percentage of correct answers before and after the intervention belonged to question 9 (the most important oral complication of DM being the altered sense of taste).

As shown in Table 4, the frequency of tooth brushing twice a day or more in physician-aid group increased from 27.5% at baseline to 50% after the intervention, which showed maximum change compared with other two groups (P < 0.001). The frequency of dental flossing once a day or more increased in all three groups after the intervention (P = 0.01). The greatest improvement in dental flossing once a day or more occurred in the social media group (P = 0.01).

**Table 4:** Oral health behavior change after the intervention compared with baseline

Groups	Tooth brushing		Flossing		Smoking	
	Baseline	After intervention	Baseline	After intervention	Baseline	After intervention
Physician-aid	27.5%	50.0%	25.0%	36.8%	85.0%	84.2%
Researcher-aid	40.0%	55.6%	45.0%	61.1%	72.5%	75.0%
Social media	37.5%	45.9%	47.5%	70.3%	68%	64.9%
p-value*	0.46	0.71	0.07	**0.01**	0.17	0.15

Bold: p-value < 0.05

*Chi-square tests

### Effect of the intervention on HbA1c

Comparing mean level of most recent HbA1c showed a reduction after the intervention compared with baseline in all three groups. However, this reduction was not statistically significant (P = 0.83). It did not reach below 7% in any of the three groups. Thus, the three groups had no significant difference in level of HbA1c after controlling for the effect of its baseline value by ANCOVA (P = 0.15, Table 5).

**Table 5:** Comparison of the mean level of most recent HbA1c* at baseline and after the intervention (n = 108)

Groups	Observed mean HbA1c		Adjusted mean HbA1c**	After the intervention _ Baseline	
	Base line	Follow up			
	Mean (SD)	Mean (SD)	Mean (SE)	Mean (SD)	p-value***
Physician-aid	7.29 (1.67)	7.37 (1.66)	7.47 (0.11)	-0.084 (0.65)	0.43
Researcher-aid	7.51 (1.36)	7.48 (1.30)	7.37 (0.12)	0.039 (0.77)	0.77
Social media	7.40 (1.54)	7.25 (1.65)	7.24 (0.11)	0.154 (0.69)	0.18
p-value	0.83	0.83	0.37		

*Hemoglobin A1c

**Adjusted with baseline values

***Paired t-test at alpha less than 0.05

## Discussion

This study aimed to assess the effect of educational interventions on oral health knowledge of diabetic adults in Tehran, Iran. Considering present evidence regarding the mutual correlation of periodontal disease and DM, effective behaviors to prevent and control periodontal disease not only improve oral health of diabetic patients, but also promote their general health [10–12].

The findings of the current study demonstrated that educational intervention may improve persons with diabetes’ awareness of oral health. Additionally, social media education was more successful than the other two techniques in improving diabetes patients’ awareness of oral health. The current findings validated the greatest effectiveness of educational interventions for enhancing oral self-care practices in diabetic people, such as brushing and flossing. The baseline findings showed that the patients with a minimum daily toothbrushing frequency of twice or more, and daily use of dental floss had better HbA1c status [31], which was in agreement with results were reported by Farahat et al., in 2016 in Yazd city, Iran, and Su et al., in 2016 in China [32, 33].

A previous study revealed that only one-third of participants were aware of the effects of periodontal disease on DM [31]. Thus, educational intervention can efficiently enhance oral health knowledge, and consequently promote oral health status of patients. Moreover, the frequency of oral diseases and subsequent oral health-related costs would decrease [34]. Nesha et al. [35] observed that only a tiny portion of their research group had received information on the impact of periodontal disease on DM from their endocrinologist, which is consistent with the current findings. The mean oral health knowledge score of all three groups increased after the educational intervention, which was in agreement with the results of studies conducted in Iran [36, 37], and Thailand [38].

Evidence shows that social media have a profound impact on improvement of oral self-care behaviors, and in terms of easy access, availability, and cost-effectiveness, they decrease social inequalities in accessing the healthcare information [39–42]. Similar to the last research, this one found that social media interventions for education were more successful than the other two approaches. As a result, experts advise diabetic patients to utilize social media to enhance their self-management and blood sugar control abilities [25, 43]. However, physicians should not use the social media as an alternative for the time they allocate to each patient. Instead, they should recommend them as an adjunct to spread oral health information in addition to educate and motivate their patients during their office visits [44].

The present results confirmed the optimal efficacy of educational interventions for enhancement of oral self-care behaviors, such as toothbrushing and dental flossing in diabetic adults, which was consistent with the findings of previous studies [36, 38, 45].

The level of HbA1c in all three groups decreased after the intervention, but not significantly. Kapellas et al., in 2017 in Australia did not report a significant reduction in HbA1c level in diabetic patients at 3 months after non-surgical periodontal therapy by scaling and root planing. Thus, Bakhshandeh et al., in 2010 in Tehran did not report a significant reduction in HbA1c level after an educational intervention using an oral self-care brochure and educational protocol in the diabetic patients 1 year after the intervention. It is believed that reduction in HbA1c level is multifactorial, and depends on several factors, such as exercise, nutrition, and therapeutic interventions [46, 47]. However, some others reported a significant reduction in HbA1c level, which can be in terms of non-surgical periodontal therapy by scaling and root planing and longer follow-ups [48, 49].

Future studies are recommended to use other social media platforms such as Instagram, YouTube, and Twitter. Besides, based on the study in a private office in Tehran, it is better to conduct further studies in hospitals and public health centers in several different cities. The present intervention lasted 3 months due to limited budget and time restrictions. Future interventions over longer periods of time are required since they may be able to significantly change the level of HbA1c. The insurance system, economic considerations, and other elements that have an impact on the analyzed associations but were not discussed in this research are suggested to be clarified in further investigations.

## Conclusion

In total, the present results showed that educational interventions enhanced oral health knowledge of diabetic adults. The community may effectively use educational interventions via social media to assist diabetes individuals avoid oral and dental issues, periodontal disease, and help lower their HbA1c.

### Suggestions

Considering the efficacy of this designed educational method through the social media, it should be recommended for its widespread use by the healthcare system. Thus, future same studies are recommended to focus on the oral health of patients with diabetes since their oral health affects their systemic health as well.

## Data Availability

The data that support the findings of this study are available from the corresponding author upon reasonable request.

## Abbreviations

DM: Diabetes mellitus
HbA1c: Hemoglobin A1c
